# Comparison of scoring systems for patients with head injury presenting to the emergency department

**DOI:** 10.1007/s00068-024-02589-6

**Published:** 2024-06-28

**Authors:** Alihan Eryilmaz, Taner Sahin

**Affiliations:** 1 Emergency Medicine Clinic, Inegol State Hospital, Bursa, Türkiye Turkey; 2 Department of Emergency Medicine, University of Health Science Kayseri Medicine Faculty, Kayseri, Türkiye Turkey

**Keywords:** Head injury, Scoring systems, Emergency department, CT scan

## Abstract

**Purpose:**

The present study aimed to compare the National Emergency X-Radiography Utilization Study II (NEXUS-II), New Orleans Criteria (NOC), Canadian Computed Tomography (CT) Head Rule (CCTHR) scoring systems, and Advanced Trauma Life Support (ATLS^®^) 10th edition algorithm in patients with head injury presenting to the emergency department and to investigate the effectiveness of these scoring systems in determining injury severity and the need for cranial CT scanning.

**Methods:**

This prospective and observational study was conducted in a tertiary care emergency medicine clinic. The study included 794 adult patients who had a Glasgow Coma Scale (GCS) score ≥ 13, and were considered as having minor head injury. Patients included in the study were categorized as having low or high risk according to the CCTHR, NOC, NEXUS-II scores, and ATLS algorithm.

**Results:**

The mean age of the patients was 40.7 ± 18.7 years, and 592 (74.6%) were male. The proportion of patients considered as having high risk was 27.7%, 84.8%, and 34.5% according to CCTHR, NOC, and NEXUS-II, respectively. According to the ATLS, 14.7% and 14.1% of the patients were considered at medium risk and high risk, respectively. CT scanning was performed in 757 (95.3%) patients, and pathologic findings were detected in 18 patients (2.3%). NOC in contrary showed a sensitivity of 100% but a specificity of 15.6%.

**Conclusion:**

In our region, there was no significant difference among the CCTHR, NEXUS-II systems, and ATLS algorithm regarding the accuracy of pathological findings in patients with head injury; any of these systems can be used in clinical practice and determining CT scan necessity. Although the sensitivity of the NOC system is very high, it has been observed that its low specificity may lead to a large number of unnecessary CT scans, which may increase the patient-based cost and waiting time in the emergency department.

## Introduction

Traumatic injuries cause 4.4 million deaths worldwide every year and account for approximately 8% of all deaths [1]. Head injury is the leading cause of trauma-related death and disability. Mortality and disability rates increase with the increase in the severity of traumatic injury [2].

Patients with head injury usually present to emergency departments (ED) of hospitals, where there is a frequent use of diagnostic imaging techniques. The Glasgow Coma Scale (GCS) is the most widely used classification system in clinical practice to determine head injury severity and the type of interventions to be taken [3]. Computed tomography (CT) is the most common imaging procedure used for diagnosis, treatment, and follow-up of patients with head injury. However, imaging procedures are ordered too frequently for patients with minor head injury in the ED, and unnecessary CT scans place a significant burden on the healthcare system, leading to increased waiting times and length of stay in the ED and costing approximately $1 billion annually [4]. This has led to the development of various scoring systems to determine indications for imaging in patients with minor head injury. The main purpose of these scoring systems is to determine the severity of injury and need for imaging [5].

The aim of the present study was to compare the performance of different scoring systems including National Emergency X-Radiography Utilization Study II (NEXUS-II) [6], New Orleans Criteria (NOC) [7], Canadian Computed Tomography Head Rule (CCTHR) [8], and Advanced Trauma Life Support (ATLS®) 10th edition algorithm [9] and to determine the sensitivity and specificity of these scoring systems in detecting the severity of head injury.

## Methods

### Power analysis

Prior to the study, the sample size was calculated based on the number of patients who presented to the ED with head injury. Power analysis was performed using G*Power (Version 3.1.9.7 for Windows). Thus, in a single-sample design, a minimum of 199 patients was determined to be sufficient for the study with 80% power, a 5% margin of error, and an effect size of 0.20%.

### Study population

This study was performed in line with the principles of the Declaration of Helsinki. Approval was granted by the Ethics Committee of Kayseri City Hospital Clinical Ethics Committee with the date and number 04.03.2021/11. Informed consent was obtained from all individual participants included in the study.

The study is a prospective and observational study conducted in Kayseri City Hospital’s tertiary care emergency medicine clinic over a 3-month period between March 01, 2020 and June 01,2020. Initially, 1,021 patients were selected to improve the accuracy of data, to determine which of the scoring systems would be more useful in patients with minor head trauma, and to enhance the reliability of the study. Later, the study excluded patients who did not consent (12 patients), patients < 18 years of age (146 patients), pregnant patients (14 patients), and patients who had a GCS score < 13 and were unstable (55 patients). Finally, based on the exclusion criteria, a total of 794 consenting male or female patients aged ≥ 18 years, with a GCS score ≥ 13, who were conscious, without an open skull fracture, and with stable vital signs and considered having minor head injury were included in the study.

### Local data analysis

Demographic characteristics (age, sex, and nationality), mechanisms of trauma, mode of presentation, examination findings, imaging results, length of follow-up, and clinical outcomes of the patients were recorded. All patients included in the study were categorized into low- or high-risk groups according to the CCTHR, NOC, NEXUS-II scoring systems, and ATLS algorithm [6–9]. Patients classified as having low risk based on their score did not undergo cranial CT initially but were monitored in the ED. Patients showing signs of increased intracranial pressure (ICP) syndrome such as nausea, vomiting, altered consciousness, and seizures during observation proceeded to undergo cranial CT. Patients who were considered as having high risk according to any of the scoring systems directly underwent cranial CT. After follow-up by the emergency physician, patients who had no cranial abnormality on CT, had stable vital signs, and had no signs of increased ICP syndrome were discharged after consultation with the neurosurgeon and were informed on signs of increased ICP syndrome that require emergency care. Discharged patients were not followed up at home.

### Statistical analysis

The study data were tabulated using descriptive statistics. Continuous (numerical) variables were summarized as mean ± standard deviation or median (minimum–maximum) values depending on the distribution. Categorical variables were summarized as number and percentage. Numerical variables were checked for normality of distribution using the Kolmogorov–Smirnov test. Decision-making criteria were evaluated based on sensitivity, specificity, accuracy, and negative and positive predictive values. Statistical analyses were performed using Jamovi (Version 1.6.7.0) and JASP (Version 0.14) programs. Statistical significance (p-value) was set at 0.05.

## Results

### Study population

The study included 794 patients with a mean age of 40.7 ± 18.7 years. Of the patients, 592 (74.6%) were male and 202 (25.4%) were female. Falling on the same level was the most common reason for presenting to hospital (201 patients; 25.3%) (Table 1).

**Table 1:** Demographic and clinical characteristics of patients

**Age (years)** ^a^	40.7 ± 18.7
≥ 65	102 (12.8)
< 65	692 (87.2)
**Sex** ^b^	
Female	202 (25.4)
Male	592 (74.6)
**Type of injury** ^b^	
Fall on the same level	201 (25.3)
In vehicle traffic accident	167 (21)
Non-vehicular traffic accident	26 (3.3)
Nose injury	23 (2.9)
Head hit by a falling object	34 (4.3)
Head hit on same level	94 (11.8)
Assault	156 (19.6)
Falling from a height	93 (11.7)
**Mode of arrival** ^b^	
Walk-in	319 (40.2)
Ambulance	475 (59.8)

^a^, mean ± standard deviation; ^b^, n (%)

### Results of injury scoring systems

Risk groups were analyzed according to scoring systems. Thus, 27.7%, 84.8%, and 34.5% of patients were assessed as high risk according to CCTHR, NOC, and NEXUS-II criteria, respectively. The proportions of patients based on the low-, moderate-, and high-risk differentiation of ATLS algorithm were 71.2%, 14.7%, and 14.1%, respectively. In the present study, CT was scheduled for both medium- and high-risks patients, which meant that patients classified as having medium risk according to the ATLS algorithm were incorporated into the high-risk group, increasing the total proportion of high-risk patients to 28.8% (Table 2).

**Table 2:** Risk stratification of patients with head injury according to different decision-making criteria

	Number (Percentage)
**Canadian Computed Tomography Head Rule** ^a^	
Low risk	574 (72.3)
High risk	220 (27.7)
**New Orleans Criteria** ^a^	
Low risk	121 (15.2)
High risk	673 (84.8)
**NEXUS-II Criteria** ^a^	
Low risk	520 (65.5)
High risk	274 (34.5)
**ATLS-10 Algorithm** ^a^	
Low risk	565 (71.2)
Medium risk	117 (14.7)
High risk	112 (14.1)
**ATLS-10 Algorithm 2** ^a^	
Low risk	565 (71.2)
High risk	229 (28.8)

NEXUS-II, National Emergency X-Radiography Utilization Study II; NOC, New Orleans Criteria; CCTHR, Canadian Computed Tomography Head Rule; ATLS, Advanced Trauma Life Support.

^a^ATLS2: Patients in the moderate- and high-risk groups were combined into one single high-risk group.

Analysis of the findings showed that age ≥ 65 years and falling from an elevation > 90 cm or > 5 steps were the most common CCTHR criteria for classification into high risk seen in 101 patients (12.7%) and 91 patients (11.5%), respectively. The most common NOC finding in the present study was a lesion on the clavicle in 612 patients (77.1%). According to these findings, the NOC recommended cranial CT for 673 patients (84.8%). The most common finding in NEXUS-II criteria was scalp hematoma, observed in 199 patients (25.1%). For the ATLS algorithm, the most common finding was dangerous trauma, detected in 117 patients (14.7%) (Table 3).

**Table 3:** Proportional distribution of variables in decision-making criteria systems according to their positive status

	Number of patients (*n* [%])
**Canadian Computed Tomography Head Rule**	
Age ≥ 65 years	101 (12.7)
> 2 episodes of vomiting	9 (1.1)
Suspected depressed or open skull fracture	0 (0)
Signs of basilar skull fracture	0 (0)
Glasgow coma scale < 15 at two hours after injury	3 (0.4)
Retrograde amnesia	11 (1.4)
Pedestrian hit by a motor vehicle	15 (1.9)
Occupant ejected from a motor vehicle through the windshield	16 (2)
Falling from an elevation > 90 cm or > 5 steps	91 (11.5)
**New Orleans Criteria (NOC)**	
Age > 60 years	120 (15.1)
Headache	179 (22.5)
Vomiting	9 (1.1)
Intoxication (drugs/alcohol)	5 (0.6)
Persistent anterograde amnesia	2 (0.3)
Signs of trauma above the clavicle	612 (77.1)
Seizure	5 (0.6)
**NEXUS-II Criteria**	
Age ≥ 65 years	101 (12.7)
Neurologic deficit	2 (0.3)
Coagulopathy	10 (1.3)
Evidence of significant skull fracture	0 (0)
Scalp hematoma	199 (25.1)
Abnormal behavior	6 (0.8)
Persistent and severe vomiting	9 (1.1)
Altered level of alertness	17 (2.1)
**ATLS Algorithm**	
Age ≥ 65 years	101 (12.7)
> 2 episodes of vomiting	9 (1.1)
Suspected open or depressed skull fracture	0 (0)
Signs of basilar skull fracture	0 (0)
Glasgow coma scale < 15 at two hours after injury	3 (0.4)
Anticoagulant use	29 (3.7)
Loss of consciousness for > 5 min	14 (1.8)
> 30 min before impact	4 (0.5)
Dangerous trauma	117 (14.7)

NEXUS, National Emergency X-Ray Utilization Study; ATLS, Advanced Trauma Life Support.

### Clinical and radiologic characteristics

Cranial CT was the most common imaging technique and was performed on 757 patients (95.3%) in the study group. Imaging showed pathologic findings in 18 patients (2.3%). Three of these patients had normal initial imaging results and received a diagnosis after repeat imaging was performed for clinical necessity. Sixteen patients (2%) underwent repeat imaging. Two patients who underwent repeat imaging were hospitalized. A total of 19 patients (2.4%) were hospitalized and one patient (0.1%) underwent surgery for shift in subdural hematoma (Table 4).

**Table 4:** Distribution of characteristics during treatment and follow-up

	Value
**Imaging technique** ^‡^	
Direct radiography	36 (4.5)
CT	757 (95.3)
MRI	1 (0.1)
**Imaging result**	
Normal, yes	776 (97.7)
Fracture	8 (1)
Subarachnoid hemorrhage	5 (0.6)
Epidural hematoma	6 (0.8)
Subdural hematoma	4 (0.5)
Intraparenchymal hematoma	1 (0.1)
Contusion	1 (0.1)
Diffuse cerebral edema	0 (0)
**Monitoring duration (h)** ^β^	2 [0–10]
**Consultation status**, yes^‡^	37 (4.7)
**Need for repeat imaging**, yes^‡^	16 (2)
**Repeat imaging result** ^‡^	
Positive	3 (18.8)
Negative	13 (81.3)
**Outcome** ^‡^	
Discharge	775 (97.6)
Hospitalization	18 (2.3)
Surgery	1 (0.1)

^‡^, n (%); ^β^, median [min–max]; CT, computed tomography; MRI: magnetic resonance imaging.

### Outcome

According to imaging results, the CCTHR had 88.9% sensitivity and 73.7% specificity in detecting the presence of any pathologic finding. The NOC had the highest sensitivity (100%), and its specificity was 15.6%. The CCTHR yielded the highest accuracy in predicting cranial pathology (74.1%). In contrast, NEXUS-II and ATLS systems had an accuracy rate of 67% and 72.9%, respectively. Based on the imaging results, the CCTHR had 89.5% sensitivity and 73.8% specificity in predicting hospitalization (*n* = 19). Of all the decision-making criteria tested, the CCTHR yielded the highest accuracy (74.2%) (Table 5).

**Table 5:** Validity analysis of decision-making criteria in predicting the presence of any pathology and hospitalization

Prediction										
	Sensitivity(%)		Specificity(%)		Accuracy(%)		NPV(%)		PPV(%)	
	Pathology	Hospitalization	Pathology	Hospitalization	Pathology	Hospitalization	Pathology	Hospitalization	Pathology	Hospitalization
**CCTHR**	88.9	89.5	73.7	73.8	74.1	74.2	99.7	99.7	7.3	7.7
**NOC**	100.0	100.0	15.6	15.6	17.5	17.6	100.0	100.0	2.7	2.8
**NEXUS-II**	83.3	84.2	66.6	66.7	67.0	67.1	99.4	99.4	5.5	5.8
**ATLS**	88.9	89.5	72.6	72.6	72.9	73.0	99.6	99.6	7.0	7.4

CCTHR, Canadian Computed Tomography Head Rule; NOC, New Orleans Criteria; NEXUS, National Emergency X-Ray Utilization Study; ATLS, Advanced Trauma Life Support; NPV, negative predictive value; PPV, positive predictive value.

## Discussion

Traumatic injuries remain a serious public health problem worldwide and the most common cause of death in people aged 15–44 years. Head injuries are the most common cause of trauma-related death [2]. They include a wide range of injuries, from mild head injuries to traumatic brain injury (TBI). TBI causes a significant number of deaths and temporary or permanent disability every year [10]. In the USA, TBI contributed to 63,262 deaths in 2020 [11]. Head injuries also result in significant medical expenditures and loss of productivity [12, 13].

Trauma scoring is the cornerstone for determining the severity of injuries and the need for imaging. EDs have high numbers of patient admission and very frequently make use of CT and/or magnetic resonance imaging (MRI) examinations for the detection of cranial pathologies [5, 14]. However, standardization still remains to be achieved worldwide in determining the severity of injuries and the need for imaging in these patients. Indeed, imaging is used in each clinic at varying frequencies depending on experience, equipment, and capacity. A number of trauma scoring systems have been developed to ensure standardization. In our study, we used the four most commonly used scoring systems—CCTHR, NOC, NEXUS-II, and ATLS algorithm —and investigated similarities and differences among these systems in predicting cranial pathologies and patient hospitalization.

In the present study, CT was the most common imaging technique performed; it was performed on 757 patients (95.3%), and pathologic findings were detected in 18 patients (2.3%). While all patients who underwent X-ray were discharged, one patient required MRI and was discharged after follow-up. Further, 16 patients (2%) required repeat imaging because of symptoms of increased ICP syndrome that developed during follow-up. Of patients who underwent repeat imaging, two required hospitalization. The rate of pathologic findings on CT was reported to be 6.7% by Saboori et al. and 9.1% by Alzuhairy [15, 16]. In the present study, the number of patients classified into low risk by the scoring systems was relatively higher, but the orders for imaging were quite high. In contrast, the rate of pathologic findings on CT was lower than that in other studies. This may have been caused by the large number of unnecessary CT scans ordered without indication because of demands of patients and family caregivers and physicians’ sense of defensive medicine and fear of malpractice.

In the present study, the CCTHR yielded the highest accuracy in predicting cranial pathology at a rate of 74.1% among the 794 patients with head injuries. The CCTHR was more accurate in predicting each pathology than the other criteria. The overall accuracy rate provided by the ATLS-10 algorithm (72.6%) was very similar to that of the CCTHR. Stiell et al. reported that the CCTHR had a specificity and sensitivity of 100% and 65.6%, respectively, values similar to those in the present study [17]. Zyluk et al. reported a specificity of 100% and a sensitivity of 53% for the CCTHR [18]. Although the NOC identified all patients with pathologies, its overall specificity rate remained at 15.6%. Stiell et al. reported a specificity of 12.1%, consistent with that in the present study [17]. This may have led to a large number of unnecessary CT scans, increasing patient-based costs and waiting time in the emergency department. The NOC system may have led to a large number of unnecessary CT scans, increasing patient-based costs and waiting time in the emergency department.

In our study, we found the sensitivity, specificity, and accuracy of the CCTHR in detecting any pathologic finding (*n* = 18) to be 88.9%, 73.7%, and 76.8%, respectively. In a Canadian study, Stiell et al. found the CCTHR to have a sensitivity of 100%, a specificity of 50.6%, and an accuracy of 76% [19]. If the patients included in the present study had been assessed exclusively with the CCTHR, 16 patients would have been initially identified as having a pathologic finding and two patients would have received a diagnosis later on through follow-up CT. Similarly, if the need for imaging had been guided by the CCTHR results, only 202 patients would have received CT scans instead of 757. These rates can be taken as evidence that clinicians should rely on trauma scoring systems to determine the need for imaging and to detect pathologic findings after trauma.

Further, the number of patients who underwent CT scanning in the present study (775) is higher than what was recommended by the NOC (675 patients), the scoring system that recommended CT the most; this was likely due to preferences of the clinician and the demand and pressure of patients’ family caregivers. This may have caused a large number of unnecessary CT scans for patients, increased patient-based costs, and increased waiting time in the emergency department. This suggests that decisions concerning patients with head injury are determined by scoring systems and influenced by preferences of clinicians, patients, or family caregivers. Nevertheless, if imaging decisions had been guided by a scoring system alone, 10% fewer patients would have undergone CT, which would have reduced radiation exposure and hospital costs. Previous studies have yielded similar results concerning the utility of scoring systems [19, 20]. With the CCTHR, this rate goes up to 58%. Stiell et al. compared the CCTHR and NEXUS-II and found a specificity of 90.7% and a sensitivity of 36.8%, which is not significantly different than what was found in the present study [19]. It was concluded that CCTHR may be somewhat more useful. Mower et al. also recommended the CCTHR based on a slight but statistically insignificant difference [21].

In our study, the majority of the 794 patients with head injury were male (74.6%). This proportion is similar to that reported by Hardman et al. (78%) [22]. Kasmaei et al. also reported the majority of patients to be male (81.8%) [23]. Mirzai et al. found a similar sex distribution [24]. High prevalence of head trauma among men than women can be attributed to various factors; men work in physically demanding and more labor-intensive jobs, are more frequently involved in assaults, or drive cars more frequently than women. The mean age of the patients in the present study was 40.7 years. Kasmaei et al. reported a mean age of approximately 38.7 years [23]. The majority of the working population worldwide is composed of young or middle-aged people, and this rate is proportionally higher in underdeveloped and developing countries. Thus, this group is the most vulnerable to head injury. The average age in our study, which included patients aged > 18 years, may be attributed to the proportion of the working and active population.

Analysis of the scoring systems used in the present study in terms of predicting hospitalization showed that the highest sensitivity was achieved by the NOC (100%) and the highest accuracy was achieved by the CCTHR (74.2%). Of all the patients, 775 (97.6%) were discharged from the ED and 19 patients were hospitalized, while one patient required surgery. Thirty-seven patients (4.7%) required neurosurgical consultation. These rates may be evidence that most patients do not need CT. Traumatic head injuries are classified into two: (1) primary injury, the injury that occurs during or shortly after the trauma; and (2) secondary injury, a clinical condition that develops over time after the initial injury [12]. The clinician’s goal for a patient presenting with head injury is to ensure urgent diagnosis and treatment of the primary injury and prevent secondary injury. In our study, repeat imaging was performed on 16 patients (2%). Two patients who underwent repeat imaging were hospitalized. Marques et al. reported that when patients with head injury on warfarin underwent repeat CT scans, 1.66% of them exhibited intracranial hemorrhage on control CT [25]. We believe that cranial CT may be useful in elderly patients with head injury and a history of anticoagulant use, even in the absence of any known trauma based on reports from the patient with impaired consciousness or caregivers, as CT in these patients can help detect intracranial pathologies such as acute or chronic hemorrhage and reveal a forensic event such as abuse of the elderly.

In the present study, the most common reasons for seeking care were falling on the same level (25.3%), followed by traffic accidents (21.0%), and assault (19.6%). In a study by Kasmaei et al., motorcycle accidents were reported to account for a larger number of admissions, which may be due to the greater use of motorcycles in Iran compared to Türkiye [23]. Similarly, Ro et al. reported that traffic accidents were responsible for a larger number of admissions compared with those from falls [26]. In the present study, the most significant factor for positive CT findings was age ≥ 65 years. This appears to indicate an increase in the number of head injuries and an increased likelihood of pathologic findings on imaging with advanced age.

We suggest the addition of a new criterion to the existing scoring systems as an indication for CT: object falling on the head of a worker not wearing a hard hat. This situation is common in our region among patients who sustain an occupational accident. Additional items can be added to the scoring systems based on the mass of the object falling on the head and the distance it falls from. Further, we suggest that drug or alcohol intoxication should be included in the scoring systems as an indication for CT as such patients are unable to fully express themselves.

### Limitations and strengths

There are some limitations to the present study including its single-center design, exclusion of patients with aged < 18 years, and CT scans performed without any indication by the scoring systems, mainly due to demands of patients’ caregivers.

However, the study also has some strengths: it was prospective, included a larger number of patients than comparable studies, and was the first to compare the ATLS algorithm with the other scoring systems.

## Conclusion

It would be useful to combine clinical examination with at least one scoring system in determining the severity of the head injury and the need for cranial CT scanning. Scoring systems may be useful in safely reducing the number of unnecessary CT scans and reducing waiting hours and costs in emergency departments. In our study, NOC, CCHR, NEXUS-II scoring systems, and ATLS-10 algorithm were used in patients with head trauma before cranial CT scan. Then, the accuracy rates in detecting cranial pathology were compared. As a result of the study, it was determined that CCHR, NEXUS-II scoring systems, and ATLS-10 algorithm had high accuracy rates in predicting patients with pathological findings and determining hospitalization. In our region, there was no significant difference among the CCTHR, NEXUS-II scoring systems, and ATLS algorithm in terms of accuracy of pathological findings in patients with head injury; any of these systems can be used in clinical practice in determining the need for CT scan. ATLS algorithm predict head injury severity and pathology at a rate similar to the CCTHR. Also, despite the specificity of the NOC system was very high, its sensitivity was very low. For this reason, it was thought that the NOC system may have led to a large number of unnecessary CT scans, increasing patient-based costs and waiting time in the emergency departments.For this reason, we do not recommend the use of the NOC system in determining the need for CT scanning in patients with head trauma.

New criteria need to be included in the scoring systems for patients with specific conditions—patients who have undergone surgery, obese patients, patients with a low baseline GCS score, patients hit on the head by an object falling from a height, and patients under the influence of alcohol or substance. This issue warrants further studies. Patients on anticoagulants who have sustained a head injury should certainly undergo CT imaging.

## Data Availability

Data for this article can be requested from the corresponding author via email.
